# Influence of Chitosan on the Viability of Encapsulated and Dehydrated Formulations of Vegetative Cells of Actinomycetes

**DOI:** 10.3390/polym16192691

**Published:** 2024-09-24

**Authors:** María Elena Mancera-López, Josefina Barrera-Cortés

**Affiliations:** Biotechnology and Bioengineering Department, Center for Research and Advanced Studies of the National Polytechnic Institute, Zacatenco Unit, Mexico City 07360, Mexico; elenamlopez@hotmail.com

**Keywords:** biologic control, alginate chitosan composites, phytopathogens, airflow dehydration

## Abstract

This study focuses on developing an encapsulated and dehydrated formulation of vegetative actinobacteria cells for an efficient application in sustainable agriculture, both as a fungicidal agent in crop protection and as a growth-stimulating agent in plants. Three strains of actinobacteria were used: one from a collection (*Streptomyces* sp.) and two natives to agricultural soil, which were identified as S3 and S6. Vegetative cells propagated in a specific liquid medium for mycelium production were encapsulated in various alginate–chitosan composites produced by extrusion. Optimal conditions for cell encapsulation were determined, and cell damage from air-drying at room temperature was evaluated. The fresh and dehydrated composites were characterized by porosity, functional groups, size and shape, and their ability to protect the immobilized vegetative cells’ viability. Actinomycetes were immobilized in capsules of 2.1–2.7 mm diameter with a sphericity index ranging from 0.058 to 0.112. Encapsulation efficiency ranged from 50% to 88%, and cell viability after drying varied between 44% and 96%, depending on the composite type, strain, and airflow. Among the three immobilized and dried strains, S3 and S6 showed greater resistance to encapsulation and drying with a 4 L·min^−1^ airflow when immobilized in coated and core-shell composites. Encapsulation in alginate–chitosan matrices effectively protects vegetative actinobacteria cells during dehydration, maintaining their viability and functionality for agricultural applications.

## 1. Introduction

Phytopathogens, including fungi, bacteria, nematodes, and insects, pose significant threats to a wide range of agricultural crops, leading to substantial financial losses for farmers or high costs associated with their control. Among these phytopathogens, fungal microorganisms are the primary contributors to economic losses in agriculture [1,2]. While chemical fungicides are effective in controlling phytopathogenic fungi, biological control using antagonistic microorganisms is a more environmentally sustainable alternative. This approach addresses the issues associated with chemical persistence and their detrimental effects on native soil microbial flora and human health [3]. Biological control typically involves formulations such as powders, granules, and suspensions (both aqueous and oily), incorporating spores as the biological agent [4]. Spores, being the resistant phase of many microorganisms, play an important role in this process. However, not all microorganisms produce spores. For those that do not, immobilization in polymeric matrices presents a promising alternative. This method provides an appropriate microenvironment for microorganism conservation and offers physical and chemical protection against potential predators and extreme environmental conditions [5]. Among the various commercially available polymers and biopolymers, alginates are the most commonly used for immobilizing microbial strains due to their non-toxic, biodegradable, and highly compatible properties [6].

Alginate is a water-soluble anionic polysaccharide composed of alternating blocks of α-L-guluronic acid and β-D-mannuronic acid residues, linked by 1–4 glycosidic bonds and featuring free hydroxyl (–OH) and carboxyl (–COOH) groups. The ratio of guluronic to mannuronic acids determines the properties of alginate gels: those with high guluronic acid content form strong but brittle gels, while those rich in mannuronic acid produce weak, elastic gels that are stable through freezing and thawing processes [7]. Microbial strain encapsulation in polymeric materials is a proposed strategy to protect microorganisms from stressful conditions that lead to the loss of viability [8]. Among various encapsulating materials, alginate is widely used due to its safety and its ability to promote microorganism propagation [9]. The high porosity of alginate gels facilitates mass transfer from the inside of the capsule to the outside, aiding in the release of active agents. However, this mass transfer can also lead to the loss of microbial strain viability [10]. Combining alginate with other biopolymers, such as chitosan, helps control the porosity of alginate gels [11].

Chitosan is a biodegradable and biocompatible cationic biopolymer that forms gels with low mechanical resistance at acidic pH. Combining chitosan with other biopolymers that interact with its amino (–NH_2_) and hydroxyl (–OH) groups results in more mechanically resistant gels [12,13]. The antimicrobial properties of chitosan come from its positive charge, and when combined with alginate, it can be used to immobilize microbial cells [9,14]. The formation of the chitosan–alginate complex depends on the ionic charge density of the biopolymers [15]. However, biopolymers like chitosan, which are soluble in acidic pH, may bind differently to alginate based on changes in pH. Gel stability to pH changes and interactions between the carboxylic groups of alginates and the amino groups of chitosan are critical for the effective encapsulation of microorganisms. Variations in the optimal pH range for polymer binding can impact the stability of the alginate–chitosan bond, affecting its capacity to release biological material and provide protection. The molecular structure and molecular weight of each biopolymer influence the properties of the alginate–chitosan network. For alginate, the ratio of guluronic to mannuronic acids is crucial, while for chitosan, the degree of deacetylation plays a key role. Studies indicate that chitosan with a high molecular weight and high degree of deacetylation, when combined with alginate rich in guluronic acid, enhances the stability and controlled release of the active agent within the capsules. However, a higher molecular weight also increases viscosity, potentially impacting the mixing and solubility of the biopolymers [16,17]. Adjusting the concentration of polysaccharides or the cross-linking agent allows for the creation of polymeric matrices with specific desired properties. In the alginate–chitosan mixture, alginate contributes to gel stability, while chitosan enhances stability against pH and temperature changes [14].

Juric et al. [18], who immobilized *Trichoderma viride* spores in alginate–chitosan composites, reported that encapsulation protects the biological material from biotic stress and prolongs its release process; the release profile observed by these authors was of an exponential type. Similarly, Luo et al. [19], who immobilized *Bacillus velezensis* NH-1 in alginate coated with one, two, and three layers of 0.8% chitosan, recorded high propagation rates in encapsulates stored for 65 days. These encapsulates demonstrated 100% efficiency in controlling *Fusarium oxysporum* compared with the control and the two- and three-layer encapsulates.

The possibility of manipulating the properties of alginate–chitosan biopolymers has generated significant interest for their diverse industrial applications. In the pharmaceutical and food industries, they are utilized for the controlled release of biologically active substances and as antimicrobial agents, among other uses [15,20,21]. They are being explored in medicine for tissue engineering applications [22,23]. In environmental science, they have been proposed for the remediation of contaminated systems [24]. In agriculture, they are of particular interest for developing bioinsecticides and protecting seeds from pathogens and adverse environmental conditions, such as drought and extreme temperatures [25]. In both cases, the aim is to enhance the efficiency and sustainability of agricultural practices.

Actinobacteria are microorganisms that produce a variety of metabolites and have been found to have numerous applications. In agriculture, their fungicidal and bactericidal properties and their ability to promote plant growth are particularly valued [26]. For commercial use, actinobacteria are often formulated as dehydrated spores [4]. Dehydration is a common method for preserving microbial strains [27]. When combined with immobilization in polymer matrices, this technique provides comprehensive protection that maintains the viability and functionality of microorganisms upon rehydration or during controlled release applications [28,29,30]. The polymer matrix shields the biological material from mechanical damage, osmotic stress, and adverse environmental conditions such as UV radiation, extreme pH, or fluctuating temperatures. Key factors in the encapsulation process that affect microbial cell viability over time include the initial concentration of vegetative cells, properties of the polymeric matrix (i.e., pore size, stability, and ionic charge), characteristics of the microbial strain, and capsule diameter [31].

Dehydration methods for actinobacteria include freeze-drying [32], spray drying [29]—which can result in a two-fold loss of viability when drying at 110 °C—vacuum drying, hot air-drying, fluidized bed drying, and osmotic dehydration. Each method has its advantages and disadvantages, but dehydration time and viability loss are prominent issues, particularly due to the strain’s exposure to high temperatures or extreme stress caused by high airflows [33]. This study proposes the encapsulation of vegetative cells of actinomycetes in alginate–chitosan composites. It is hypothesized that the reduction in pore size of the calcium alginate gel, with the incorporation of chitosan, will reduce drying time when using high airflows and decrease viability loss by protecting the strains from extreme environmental conditions, including UV light. The preparation method of the composite affects its porosity; therefore, three types of composites were evaluated: blended, coated, and core-shell. It is important to note that although formulations with vegetative cells have a high potential for propagation, they require careful handling to prevent damage to the cellular material [34,35]. Actinobacteria, with their versatility and ability to produce diverse bioactive compounds, remain a valuable resource for biotechnology and scientific research.

## 2. Materials and Methods

### 2.1. Chemical Substances

Sodium alginate was purchased from CIVEQ (Mexico City, Mexico). The supplier did not provide data on this polymer, but NMR analysis reported by Mancera et al. [30] indicates that it is an alginate with a high mannuronic acid content (the guluronic acid content was negligible) and a viscosity of 2000 cp. The chitosan used had a medium molecular weight (140,469 g mol^−1^) and a degree of deacetylation of 84.2 ± 0.3 mol% [36]. Chitosan was acquired from Sigma-Aldrich (Sigma Chemical Corporation, Saint Louis, MO, USA), as was the yeast extract. The solid culture medium, Czapek Dox^®^ agar, was purchased from Becton Dickinson Mexico (Mexico City, Mexico). Peptone protease was purchased from Difco (Difco Laboratories, Detroit, MI, USA). All other chemicals (reactive grade) used in this research were from the J.T. Baker^®^ brand (Fisher Scientific Mexico S. de R.L. de C.V., Mexico City, Mexico): dextrose, calcium carbonate (CaCO_3_), dipotassium phosphate (K_2_HPO_4_), magnesium sulfate heptahydrate (MgSO_4_·7H_2_O), calcium chloride (CaCl_2_), sodium hydroxide (NaOH), acetic acid (CH_3_COOH), and sodium citrate (Na_3_C_6_H_5_O_7_). Distilled water was used as a solvent.

### 2.2. Microorganisms and Culture Conditions

The present study was carried out with three strains of actinomycetes. Two of the strains (S3 and S6) were selected from a total of 6 strains isolated from an agricultural soil dedicated to corn cultivation (Nextlalpan, Mexico). The selection criterion was abundant biomass production, production of growth phytoregulators, and low or no spore production during its growth phase in liquid culture. The isolated strains were analyzed for their production of indole acetic acid and their ability to solubilize phosphates. These analyses were conducted to explore the potential application of actinomycetes in the agricultural sector for stimulating plant growth; however, the analysis was only qualitative. The third strain, identified as *Streptomyces* sp., was acquired from the National Collection of Microbial and Cell Cultures (CDBB, Mexico City, Mexico) of the Center for Research and Advanced Studies of the National Polytechnic Institute (CINVESTAV-IPN). The actinomycetes were inoculated in Petri dishes with Czapek Dox^®^ agar, for incubation (Digital Incubator Felisa FE−132D, Fabricantes Feligneo, Zapopan, Mexico) at 30 °C for 7 to 15 days or until colonies with a firm texture and mycelial branches were observed. The microbial growth of each actinomycete was used in the preparation of microbial stocks or production of inocula.

For conservation, Petri dishes exhibiting visible spore production were soaked in sterile distilled water. The resulting spore suspension was collected, filtered, and transferred into 1 mL Eppendorf tubes for storage at 4 °C.

For inoculum preparation, 0.5 cm agar cubes were cut from plates with abundant cell growth and inoculated into 125 mL Erlenmeyer flasks containing 50 mL of M2 culture medium (composition: dextrose 40 g, peptone protease 9 g, yeast extract 1 g, CaCO_3_ 6 g, K_3_HPO_4_ 1 g, MgSO_4_·7H_2_O 1 g, distilled water 1000 mL, pH 7.2), previously autoclaved (Hirayama H1CLAVE HV 50, Amerex Instruments, Lafayette, CA, USA) at 121 °C and 9.8 kPa for 15 min [37]. The flasks were incubated at 30 °C and 200 rpm (Thermo Forma 420 Incubator Shaker, Forma Scientific Inc., Marjetta, OH, USA) until abundant microbial growth was observed (approximately 72 h). The purity of the culture was verified by microscopic observation and plating on Czapek Dox^®^ agar.

### 2.3. Biomass Production

Ten milliliters of inoculum (S3, S6, and *Streptomyces* sp.) with a concentration of 5.25 × 10^8^ ± 3.5 × 10^7^ CFU mL^−1^ were added to 1 L Erlenmeyer flasks containing 400 mL of M2 culture medium. The cultures were prepared in triplicate and incubated at 30 °C and 200 rpm for approximately 4 days. Once the cells had grown to the beginning of their stationary phase, the cultures were centrifuged at 9000 rpm and 4 °C for 15 min (Sorvall 6000, Thermo Scientific, Waltham, MA, USA). The resulting biomass was resuspended in 50 mL of sterile distilled water and stored at 4 °C for later encapsulation. The purity of the culture was verified by microscopic observation and plating on Czapek Dox^®^ agar.

### 2.4. Immobilization of Actinomycetes in Calcium Alginate Beads

The actinomycetes, adjusted to a concentration of 4.2 × 10^8^ ± 9.2 × 10^7^ CFU mL^−1^ for all strains, were resuspended in 200 mL of a sterilized 1.5% (*w*/*v*) sodium alginate aqueous solution along with 200 mL of M2 medium under shaking conditions (200 rpm) [30]. This biomass-alginate-medium mixture was then dripped into 400 mL of a sterilized 0.2 M calcium chloride solution using a peristaltic pump and hypodermic needle (31 G × 8 mm and 20 G × 9.5 mm), where gelation occurred with gentle stirring (150 rpm). After one hour at 25 ± 2 °C with gentle shaking, the resulting alginate beads were separated by medium-pore filtration, washed with sterile distilled water, and stored, suspended in sterile distilled water, at 4 °C until use. Capsules were consistently produced with approximate diameters of 2–3 mm (see Section 2.11.4), determined by the needle’s orifice diameter and the fixed distance from the surface of the gelling solution. The viability of encapsulated actinomycetes was determined as colony-forming units (CFU mL^−1^), both before and after immobilization of the actinomycetes. This analysis was performed using serial dilution and plating on Czapek Dox^®^ agar plates, after solubilization of the alginate matrix with 1% (*w*/*v*) sodium citrate (6 capsules in 1 mL of sodium citrate). All experiments were conducted in duplicate using sterile materials and in a sanitized environment. The capsule shape was observed using a Zeiss SB8 Stereomicroscope (Carl Zeiss AG, Oberkochen, Germany) at 6.4×.

### 2.5. Immobilization of Actinomycetes in Chitosan Beads

Actinomycetes immobilization in chitosan was conducted using a suspension of the microbial strains (5.23 × 10^8^, 3.93 × 10^8^, and 3.43 × 10^8^ CFU mL^−1^ for S3, S6, and *Streptomyces* sp., respectively) in 100 mL of a 2% (*w*/*v*) chitosan solution, prepared with 1% (*v*/*v*) acetic acid [38]. The mixture was dripped into 300 mL of a 1 M KOH solution for gelation, under stirring at 150 rpm for 1 h. Due to the high viscosity of the 2% *w*/*v* chitosan solution, the solution was dripped using a 1 mL syringe without a needle. The formed capsules were then separated, washed thoroughly with distilled water, and suspended in sterilized distilled water for storage at 6 °C. The encapsulation process was carried out using sterilized materials and in an aseptic environment. Both capsules with and without biomass were characterized for shape, diameter, porosity, and viability of the encapsulated biomass, employing the same methods used for characterizing the calcium alginate capsules.

### 2.6. Immobilization of Actinomycetes in the Calcium Alginate–Chitosan Composite

Alginate–chitosan composites, categorized as blended, coated, and core-shell, were prepared in two stages as outlined in Table 1, following the methodology reported by Parsana et al., Gåserød et al., Bai et al., and Erdélyi et al. [11,39,40,41], with some modifications. The same procedure was followed to produce capsules and composites without biomass. The different batches of composites were separated by filtration, washed with an abundant amount of sterilized distilled water, and stored in sterilized distilled water at 4 °C. The filtrate obtained from the washing process was collected to quantify the chitosan that did not integrate into the alginate capsules (Section 2.11.1).

The composites were produced using chitosan solubilized in water at pH 3.8 and 5.5. The pH 5.5 was the maximum value allowed for chitosan solubilization. These different pH values were evaluated because actinomycetes grow in a neutral pH environment. An acidic pH could potentially affect the viability of the studied actinomycetes [42]

All experiments were conducted in duplicate using sterile materials in a sanitized environment. The shape of the formed capsules was observed under a Zeiss SB8 Stereomicroscope at 6.4× magnification.

### 2.7. Encapsulation Efficiency and Viability of Immobilized Strains

The encapsulation efficiency was determined by analyzing the count of viable actinomycetes, both free in the sodium alginate solution and immobilized in the alginate–chitosan composites. The analysis was performed in duplicate using 0.01 g capsule samples dissolved in 1 mL of sterile 1% (*w*/*v*) sodium citrate. Biomass release was conducted at room temperature with gentle stirring (150 rpm) [30]. The microbial count was determined through serial dilution and plating on Petri dishes with Czapek^®^ agar. The plates were incubated at 30 °C for 7 days. Growth (CFU mL^−1^) was recorded and reported as the mean of two replicates for each dilution. The encapsulation efficiency (%E) was measured by the survival of viable cells during encapsulation, using the following formula [11]:

(1) $$\%E=\left(\frac{N}{N_{0}}\right)100$$

where *N* = number of viable cells released from the capsules (CFU mL^−1^); *N*_0_ = number of cells as CFU mL^−1^ in the 1.5% (*w*/*v*) sodium alginate used for the formation of the alginate capsules or alginate–chitosan composites.

The stability of the composites was determined by observing changes in the shape and size of the capsules over time. A batch of 200 capsules of each composite type (coated, blended, and core-shell capsules) was placed in a 50 mL falcon tube and suspended in 25 mL of sterilized distilled water. The capsules were periodically observed every 8 days for 60 days. During this period, the falcon tubes were stored at 4 °C.

### 2.8. Dehydration of Alginate–Chitosan Composites

The drying of the immobilized biomass was conducted in 0.65 L glass cylinders (6 cm ID × 23 cm L) sterilized in an autoclave at 121 °C and 0.1 kg cm^−2^ for 15 min. The cylinders were loaded with batches of 25 ± 3 g of the different composites. The drying process involved a controlled airflow at either 4 L min^−1^ or 10 L min^−1^, fed into the cylinder at room temperature (25 ± 2.5 °C) through a porous filter for sterilization [30]. The drying process was considered complete when the relative humidity of the air exiting the dryer reached 20%, and the target capsule sizes were approximately 2 mm. This same drying process was applied to composites both with and without biomass.

The dehydrated capsules were then transferred to Petri dishes and stabilized at 30 °C for 24 h. The mass of water removed was determined by the difference in weights. The viability of the strains was assessed as CFU in 0.1 g samples of dehydrated capsules, after releasing the biomass by dissolving the polymer matrix in 10 mL of a 1% sodium citrate solution. The biomass suspension was homogenized with vortex shaking and centrifuged at 5000 rpm for 10 min. The supernatant was used for microbial count analysis through serial dilution and seeding of 100 μL aliquots on Petri dishes with Czapek^®^ agar. The plates were incubated at 30 °C for 7 days; the appearance of colonies confirmed the viability of the strain.

The drying of larger batches (125 ± 5 g) was evaluated only for the dehydrated composites that best preserved biomass viability. The drying process was evaluated in 3.5 L rotating cylinders (12 cm ID × 30 cm L) operated at 30 rpm under the same airflow (4 and 10 L min^−1^) and temperature conditions (24.5 ± 2.5 °C).

The drying of the different composites was performed in duplicate, using sterile materials and under sterile conditions. At the end of the drying process, the viability of the strain and water loss from the capsules were quantified.

### 2.9. Release of Actinomycetes from the Polymer Matrix

This analysis was conducted on 1 g batches of capsules, both for the calcium alginate matrix and the alginate–chitosan composites. The batches were immersed in 50 mL of a 0.85% NaCl solution maintained at 30 °C. After 24 and 48 h of incubation, 100 µL of supernatant was taken from each batch and plated on Petri dishes with Czapek^®^ agar. The observation of colony-forming units verified the release of immobilized biomass, which was then quantified by dry weight.

### 2.10. Exposure of Encapsulated Actinomycetes to Ultraviolet Radiation

Batches of biomass immobilized in fresh and dehydrated capsules of calcium alginate and alginate–chitosan composites were exposed to long-wavelength ultraviolet (UV) irradiation (365 nm). Irradiation was performed for 6 h in a closed chamber with a 4 W UV lamp (UVGL−25, CTR Scientific, Monterrey, N.L. Mexico) placed 7 cm from the samples. Every 2, 4, and 6 h, 0.01 g of each batch of capsules was removed and the biomass was released into 30 mL of 0.85% NaCl solution in a 125 mL Erlenmeyer flask, maintained at 30 °C. After 24 h, 100 µL of supernatant from each flask was plated on Petri dishes with Czapek^®^ agar. Microbial growth was then collected with sterile distilled water and quantified by dry weight.

### 2.11. Characterization of the Capsules

#### 2.11.1. Chitosan Content in Alginate–Chitosan Composites

The mass of chitosan in the composites was determined by measuring the difference between the initial mass of chitosan used in production and the mass of chitosan remaining in the gelling solution after filtration. Once the capsules are formed and filtered, the pH of the filtrate is abruptly increased to 11 using a 30% (*w*/*v*) NaOH solution. The filtrate is then left to stand at room temperature for 30 min, allowing the chitosan to precipitate. The precipitated chitosan is separated by filtration through a medium-pore membrane and dried at room temperature. The mass of chitosan is determined by the difference in weights.

#### 2.11.2. Porosity

The porosity of the capsules was determined using the liquid displacement method, as reported by Beltran-Vargas et al. [23]. Ethanol was chosen as the penetration medium because it does not cause shrinkage or swelling, does not dissolve polymers, and easily penetrates the pores. Each capsule sample was placed in a cylinder containing a known volume of ethanol. After 48 h, the capsules were removed, and the final volume was recorded. The porosity was calculated using the following equation:

(2) $$Porosity\left(\%\right)=\left(\frac{W_{S}-W_{0}}{\rho V}\right)100$$

where *W_S_* is the final weight of the saturated capsules, *W*_0_ is the initial weight of the capsules, *ρ* is the density of ethanol, and *V* is the volume of displaced ethanol.

#### 2.11.3. Scanning Electron Microscopy (SEM)

This analysis enabled us to identify the surface morphology of the capsules after the dehydration process. The analysis was conducted in a high vacuum environment using a JEOL JSM−6510LV scanning electron microscope (Peabody, MA, USA). To obtain sufficient contrast, the dehydrated capsules were coated with a thin gold film. Images of the capsules in representative fields were documented. The average diameter of the dehydrated capsules was determined through image analysis using ImageJ 1.46 software (public domain), developed by Tiago Ferreira and Wayne Rasband.

#### 2.11.4. Capsule Diameter and Sphericity Factor

The size of the composites was analyzed using ImageJ 1.46 software. Photographs were captured with a Zeiss SB8 Stereomicroscope at 6.4× magnification. For each sample, a minimum of 30 measurements were recorded to determine the average capsule diameter and the sphericity factor. The sphericity factor (SF) was calculated using the following equation:

(3) $$SF=\frac{d_{max}-d_{per}}{d_{max}+d_{per}}$$

where *d_max_* is the largest diameter passing through the center of the particle, and *d_per_* is the diameter perpendicular to *d_max_* passing through the center of the particle. Microcapsules with an SF less than 0.05 are considered spherical [43,44].

#### 2.11.5. FTIR Analysis

This analysis enabled the identification of the composition of the polymer matrix formed by the interaction between the biopolymer’s alginate and chitosan. The analysis was performed using a Nicolet 6700 FT–IR spectrometer (Thermo Fisher Scientific, Waltham, MA, USA). Spectra were recorded in the range of 450 to 4000 cm^−1^ with a resolution of 1 cm^−1^.

### 2.12. Statistic Analysis

The experiments were performed in duplicate, and the results reported are the mean ± standard error (S.E). Statistical analysis was performed with Minitab 18 version. Significant differences between means were determined by Tukey’s test (*α* < 0.05).

## 3. Results

### 3.1. Biomass Yield

The propagation of strains S3, S6, and *Streptomyces* sp. in liquid medium (M2) resulted in dry biomass yields of 8.01 ± 0.5 g L^−1^, 8.5 ± 0.5 g L^−1^, and 5.5 ± 1 g L^−1^, respectively, after 96 h. The microbial counts in CFU mL^−1^ were 5.23 × 10^8^, 3.93 × 10^8^, and 3.43 × 10^8^ CFU mL^−1^, respectively. Strains S3 and S6 produced abundant mycelium in the form of small, compact pellets that were easy to disperse. On solid medium, both strains formed white, dry colonies with irregular surfaces of powdery texture, and filamentous edges (Supplementary Figure S1). The average diameter of the colonies was 5–8 mm. In liquid culture (M2), strain S6 produced a dark pigment, and in solid culture (Czapek^®^ agar), the center of the colony turned dark after 7 days of growth, subsequently spreading and staining the solid substrate brown. In liquid medium (M2), the *Streptomyces* sp. strain CDBB 1232 grew in the form of heterogeneous spongy granules that were difficult to disperse.

### 3.2. Size and Shape of Capsules and Composites Containing Immobilized Biomass

**Figure 1:**
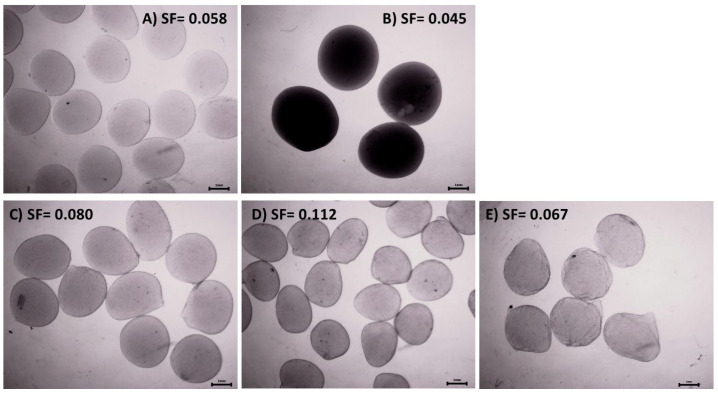
Biomass immobilized in alginate and chitosan capsules and alginate–chitosan composites prepared with chitosan solubilized at pH 3.8 (Stereomicroscope, Zeiss SB8; 6.4×). (**A**) Calcium alginate, (**B**) chitosan, (**C**) blended composites, (**D**) coated composites, and (**E**) core-shell composites. SF is the sphericity factor.

The biopolymer solutions with suspended biomass enabled the production of capsule batches of 30 ± 5 g with an average diameter ranging between 2.1 and 4 mm, as shown in Table 2. The stage at which chitosan was added during composite production influenced the consistency, size, shape, porosity, and texture of the capsules’ external surfaces. The ANOVA results indicated that capsule diameter was significantly affected by the type of polymer matrix (F = 109.817, df = 3, *p* < 0.0001, χ^2^ = 0.750) but not by pH or strain type, as per the Tukey test at α < 0.05. Table 2 also provides the shape of the capsules, expressed as the sphericity factor (SF). According to these data, only chitosan capsules met the sphericity criterion (SF < 0.05). The ANOVA results for the sphericity factor revealed significant effects solely for the type of encapsulating material (F = 3.93, df = 3, *p* = 0.01, χ^2^ = 0.102). Figure 1 illustrates the shape and corresponding sphericity factor of capsules containing immobilized biomass, prepared using alginate and chitosan biopolymers, as well as mixtures of these biopolymers in the form of blended, coated, and core-shell composites. The color of the capsules was determined by the immobilized actinomycete strain. Capsules containing the *Streptomyces* sp. and S3 strains appeared beige, while those prepared with the S6 strain exhibited a dark color, attributed to the pigment produced by the strain. Capsules with a calcium alginate core coated with chitosan developed a compact and hard structure with a medium rough surface. Blended capsules had a soft, deformable consistency with a slightly rough surface. Core-shell capsules exhibited an intermediate consistency between the previous two, with a rough surface. Capsules stored in sterile distilled water at 4 °C showed no physical changes after 6 months.

### 3.3. Encapsulation Efficiency

**Figure 2:**
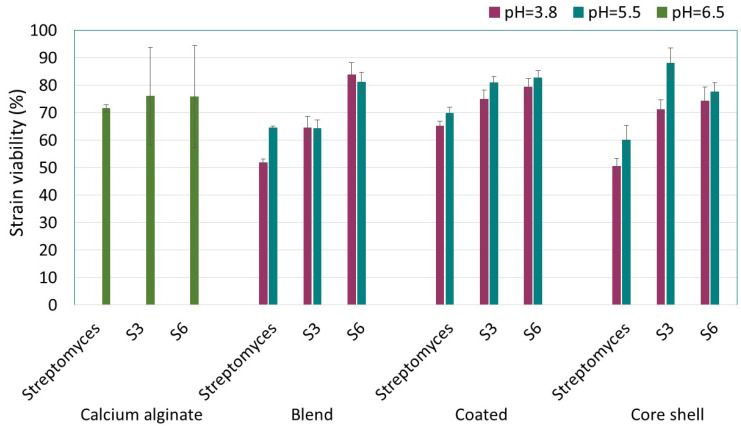
Viability (%) of actinomycetes after encapsulation in alginate–chitosan composites. Three-way ANOVA with data segmented by polymeric matrix type. According to the Tukey test for *α* < 0.05, viability is determined by strain type and pH.

The encapsulation process led to a loss of viability in *Streptomyces* sp. strains, with percentages ranging from 16% to 50%. The ANOVA of the viability of encapsulated strains, considering the effects of encapsulation type, the pH of the chitosan solution, and strain type, revealed significant differences related to strain type (F(blend capsules) = 59.459, df = 2, *p* < 0.0001, χ^2^ = 0.952; F(coated capsules) = 29.359, df = 2, *p* = 0.001, χ^2^ = 0.907; F(core-shell) = 35.285, df = 2, *p* < 0.0001, χ^2^ = 0.922) and pH (F(coated capsules) = 9.678, df = 1, *p* = 0.021, χ^2^ = 0.617; F(core-shell) = 15.377, df = 1, *p* = 0.008, χ^2^ = 0.719) according to the Tukey test for α < 0.05 (Figure 2). Among the three immobilized strains, S3 and S6 demonstrated greater resistance across the three encapsulation processes tested. The viability of these strains ranged from 71 to 79% when chitosan was solubilized at pH 3.8, and from 77 to 88% when the solubilization pH was adjusted to 5.5. These findings underscore the significance of pH, within the tested pH range, in influencing the viability of the encapsulated strains. Generally, strains encapsulated in composites prepared with chitosan solubilized at pH 5.5 exhibited less viability loss compared with those with chitosan solubilized at pH 3.8.

### 3.4. Chitosan Content in the Composites

The ANOVA of the chitosan percentage integrated into coated, blended, and core-shell composites showed significant differences due to the pH set for chitosan solubilization (F = 30.827, df = 1, *p* = 0.001, χ^2^ = 0.837) and the type of composite formed (F = 27.516, df = 1, *p* = 0.001, χ^2^ = 0.902), according to the Tukey test at α < 0.05 (Table 3). A pH of 3.8 facilitated the integration of chitosan into all three composite types, with the highest integration observed in the blend type (90.4 ± 3.3%), where chitosan was added during the alginate gelation stage. The ovoid shape of these composites could be attributed to the COO^−^ NH_3_^+^ bonds in the alginate and chitosan, respectively, available from the center of the capsule. In the coated composites, where chitosan was incorporated into the surface of soft alginate gels, capsule compaction was observed, which could explain its lower porosity (see Section 3.5). The chitosan content in the core-shell composites was higher compared with that found in those of the coated type. This result can be explained by the greater softness of the alginate gels used in the production of these composites, which were gelled with 0.03 M CaCl_2_.

### 3.5. Capsule Drying

Drying times were 9 ± 0.5 h at an airflow rate of 10 L min^−1^ and 36 ± 2 h at 4 L min^−1^. The drying time of the capsules at 30 °C took 26 ± 3 h. Recovered dehydrated capsules weighed from 0.75 to 1.25 ± 0.1 g, indicating a moisture loss of 97 ± 0.7%. Dehydrated capsules containing immobilized biomass exhibited a round shape with an average diameter of 876 ± 75 µm. In contrast, capsules without biomass collapsed, resulting in an approximate 30% reduction in diameter. Statistical analysis using the Tukey test (α < 0.05) revealed no significant differences in moisture loss due to composite type or chitosan solubilization pH (F(composite) = 1.895, df = 6, *p* = 0.152; χ^2^ = 0.448; F(pH) = 1.259, df = 1, *p* = 0.281; χ^2^ = 0.083). Increasing the batch size from 25 g to 125 ± 5 g led to a 6% decrease in strain viability and increased drying times to 26 and 54 h under air flows of 10 and 4 L min^−1^, respectively. The extended drying times likely resulted from the larger moisture volume removed.

**Figure 3:**
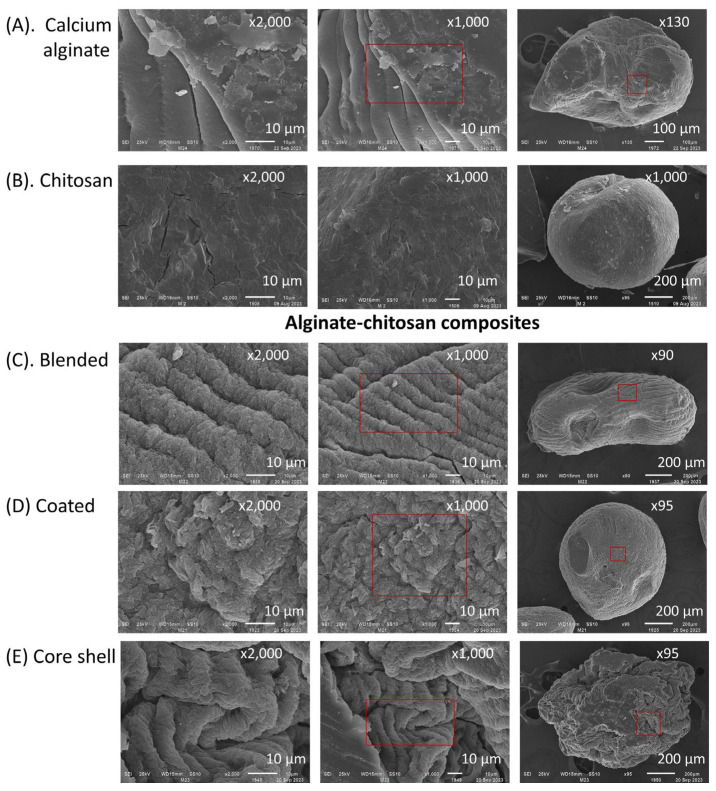
SEM of dehydrated capsules under an airflow of 10 L min^−1^. (**A**) calcium alginate (pH 6.5), (**B**) chitosan (pH 3.8), (**C**) blended, (**D**) coated, and (**E**) core-shell composites (pH 3.8).

SEM images in Figure 3 depict dehydrated capsules (using an airflow rate of 10 L min^−1^) of calcium alginate, chitosan, and three alginate–chitosan composites: blend, coated, and core-shell. Both alginate and chitosan capsules displayed a rough surface texture, with deeper channels observed particularly in the alginate capsules. In the composites, surface morphology varied significantly depending on the preparation method. For example, blend and coated composites exhibited a rough surface with a criss-crossed reticular structure. In contrast, core-shell composites showed channels with multiple folds in an open structure characterized by long and deep grooves. This distinctive morphology is likely attributable to the penetration of chitosan into a soft reticular matrix of calcium alginate.

**Figure 4:**
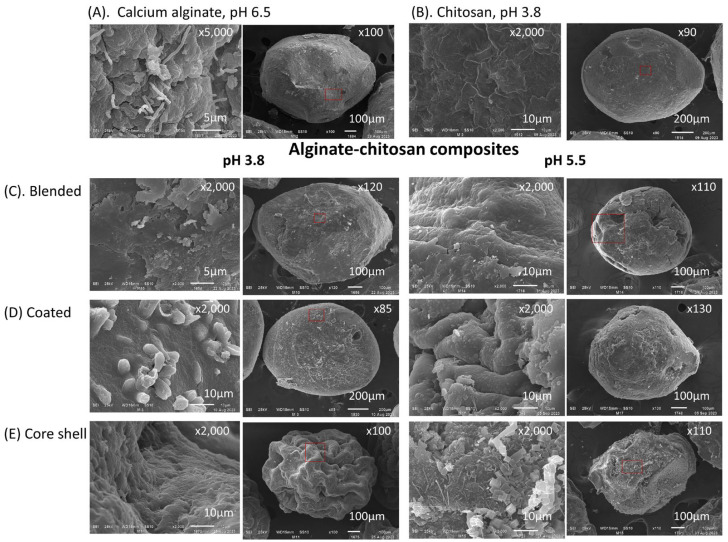
SEM analysis of actinomycete strains immobilized in calcium alginate and chitosan capsules, alginate–chitosan composites, and dehydrated under an airflow of 10 L min^−1^. (**A**) Calcium alginate capsules (pH 6.5), (**B**) chitosan capsules (pH 3.8), (**C**) blended composites (pH 3.8 and 5.5), (**D**) coated composites (pH 3.8 and 5.5), and (**E**) core-shell composites (pH 3.8 and 5.5).

Figure 4 presents dehydrated capsules of alginate and chitosan, as well as three alginate–chitosan composites produced with chitosan solubilized at pH values of 3.8 and 5.5, all containing immobilized biomass. The encapsulation of mycelium is easily verified in sodium alginate capsules and coated composites. In the former, mycelium emerges through fractures on the surface of the calcium alginate matrix (Figure 4A). In coated composites, mycelium is visible beneath the chitosan layer covering the calcium alginate matrix, illustrating the protective capability of this biopolymer (Figure 4D, pH = 3.8). Capsules formed with chitosan solubilized at pH 5.5 exhibited surfaces with embedded crystals or granules, likely due to the reduced solubility of chitosan at this pH.

**Figure 5:**
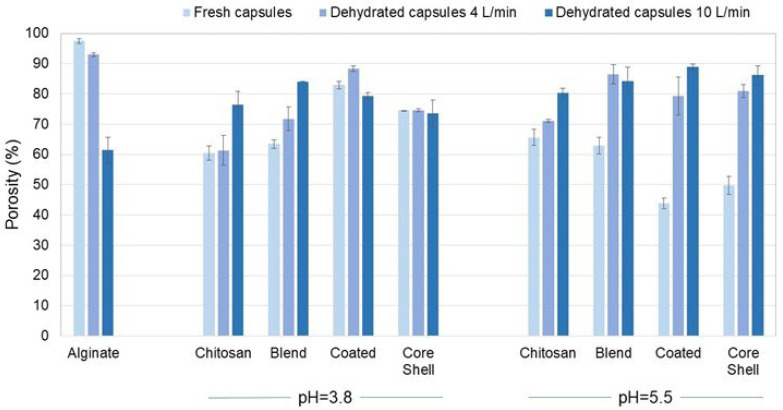
Porosity of fresh and dehydrated capsules of calcium alginate and chitosan, and their composites. The 3-way ANOVA of the polymeric matrix porosity revealed significant differences due to the polymeric matrix (F = 23.2, df = 3, *p* < 0.0001, χ^2^ = 0.720) and drying airflow (F = 30.36, df = 2, *p* < 0.0001, χ^2^ = 0.692).

**Figure 6:**
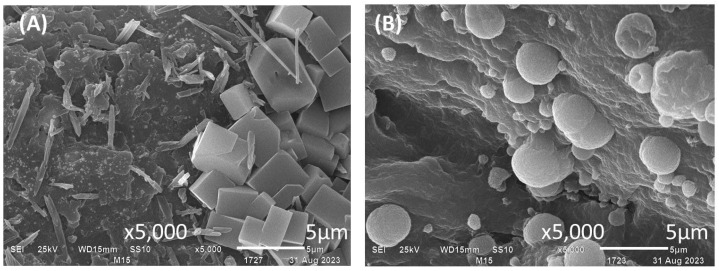
SEM of the core-shell composite produced with chitosan solubilized at pH = 5.5. (**A**) Chitosan crystals formed with calcium. (**B**) Chitosan microcapsules.

Figure 5 presents the porosity of the alginate and chitosan capsules, as well as that of their composites. ANOVA analysis of porosity, considering the effects of polymeric matrix structure, pH, and dehydration airflow, revealed significant differences due to the polymeric matrix (F = 23.2, df = 3, *p* < 0.0001, χ^2^ = 0.720) and dehydration airflow (F = 30.36, df = 2, *p* < 0.0001, χ^2^ = 0.692). Generally, changing the pH from 3.8 to 5.5 did not significantly alter the porosity of the polymeric matrix (F = 1.532, df = 1, *p* = 0.227, χ^2^ = 0.054) as indicated by Figure 5. Dehydration resulted in decreased porosity of calcium alginate, whereas the opposite results were observed for chitosan and its composites. Specifically, the lower porosity of the composites may be associated with a more compact reticular structure due to the chitosan content. Figure 6 depicts the surface of core-shell composites prepared using chitosan solubilized at pH 5.5. Previous studies have reported that chitosan can crystallize in the presence of calcium ions [45,46]. The structures observed in Figure 6B may represent chitosan microcapsules formed due to sodium ion release from alginate.

### 3.6. Viability of Encapsulated and Dehydrated Strains

**Figure 7:**
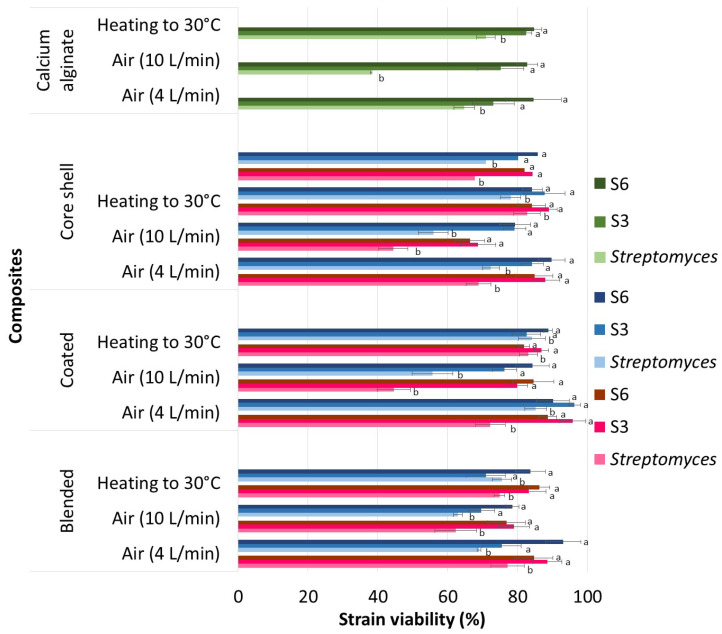
Remaining viability of encapsulated actinomycete strains subsequently dehydrated at 4 L min^−1^, 10 L min^−1^, and 30 °C. Green bars: pH 6.5, Blue bars: pH 5.5 and Pink bars: pH 3.8. The 4-way ANOVA indicated that the viability of the encapsulated and dehydrated actinomycete strains is determined by the type of encapsulating matrix, type of strain, drying airflow, and by the interaction of strain and drying airflow. Average values (±SE) that do not share an equal letter are significantly different according to Tukey’s test (*α <* 0.05).

Dehydration of actinomycete strains immobilized in alginate–chitosan composites under two different air flows (4 and 10 L·min^−1^) and heat at 30 °C resulted in the viability percentages presented in Figure 7. ANOVA of the viability of the immobilized and dehydrated strains identified significant differences due to the type of composite (F = 3.274, df = 2, *p* = 0.044, χ^2^ = 0.094), strain (F = 40.429, df = 2, *p* < 0.001, χ^2^ = 0.562), drying airflow (F = 37.115, df = 2, *p* < 0.001, χ^2^ = 0.541), and the (strain)–(drying airflow) interaction (F = 8.581, df = 4, *p* < 0.001, χ^2^ = 0.353). The dependent variable met normality assumptions as verified by the Kolmogorov–Smirnov test. Strains immobilized in composites exhibited a higher average viability (11–15%) compared with those immobilized in calcium alginate alone. Among the strains, those isolated from agricultural soil (S3 and S6) were more resistant to the drying process, showing an average viability of 13%, a percentage higher than the collection strain (*Streptomyces* sp.). Air-drying at 10 L min^−1^ resulted in average viability losses 13% greater than the other two methods (airflow at 4 L min^−1^ and heat at 30 °C), between which no significant differences were observed. Chitosan solubilization in an acidic medium (pH 3.8) did not affect the viability of the encapsulated and dehydrated strains when the pH was increased to 5.5. However, compared with the sodium alginate encapsulating solution at pH 6.5, the lower viability in the calcium alginate is attributed to its less protective properties.

The ANOVA of the viability of immobilized strains in dehydrated composites revealed a lower loss of viability in coated composites compared with blend and core-shell composites (F = 11.761, df = 2, *p* < 0.0001, χ^2^ = 0.303). In coated composites, with chitosan solubilized at pH 3.8, strain S3 exhibited the highest viability percentages: 96 ± 4% when dried with an airflow of 4 L min^−1^ and 80 ± 3% with an airflow of 10 L min^−1^. In core-shell composites, the viability of strain S3 was 88 ± 4% and 69 ± 5% under the same respective airflows. *Streptomyces* sp., the strain least resistant to drying, showed residual viabilities of 72 ± 2% and 44 ± 5% in coated composites dehydrated under airflows of 4 and 10 L min^−1^, respectively; the higher airflow significantly affected the mycelial tissue of the *Streptomyces* sp. strain. Solubilizing chitosan in an acidic medium at pH 5.5 increased the viability of *Streptomyces* sp. by 12 ± 1% in the same composite. Additionally, pH levels of 5.5 and 6.5 positively affected the S6 strain, which showed a 7% higher average viability compared with the S3 strain. Thus, the S6 strain is more resistant to drying when chitosan is solubilized at pH 5.5, as well as when the strain is dehydrated while immobilized in calcium alginate. Despite the higher viability of strain S6 at an airflow of 10 L min^−1^, the release of biomass by solubilizing the composite in 1% sodium citrate revealed fragmented and damaged mycelium.

**Figure 8:**
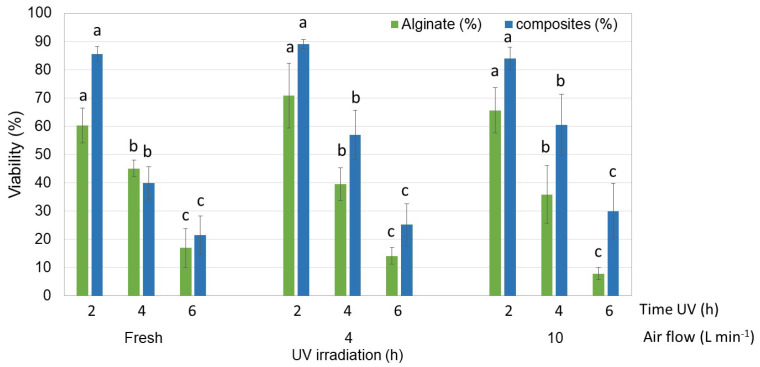
Viability of encapsulated strain S3 (fresh and dehydrated) after its exposure to 356 nm UV irradiation for 2, 4, and 6 h. The 3-way ANOVA indicated that the viability of the encapsulated and dehydrated strain was determined by the UV exposure time (F = 11.641; df = 3; *p* < 0.0001; χ^2^ = 0.492) and whether alginate was used alone or in combination with chitosan (F = 223.885; df = 2; *p* < 0.0001; χ^2^ = 0.926). Average values (±SE) that do not share an equal letter (a–c) are significantly different according to Tukey’s test (α < 0.05).

The fresh and dehydrated encapsulates of strain S3, the most resistant to the encapsulation and dehydration process, demonstrated significant resistance to 365 nm UV light. This resistance significatively varied with exposure time and the use of alginate alone or in combination with chitosan, as indicated by the Tukey test for α < 0.05 (Figure 8). Under a 2 h exposure, strain S3 encapsulated in fresh and dehydrated alginate maintained a viability above 60%, and between 84% and 89% when immobilized in the composites. In their dehydrated form, the composites provided better protection than alginate alone, increasing viability by 11% to 25%. The highest percentage was observed in composites exposed to UV for up to 6 h.

### 3.7. Preservation and Release of Biomass

The propagation levels of actinomycetes released from alginate capsules and alginate–chitosan composites, which were dehydrated and stored under refrigeration for six months, are presented in Table 4. The strain type, capsule reticular structure, airflow, and pH significantly influenced the propagation of strains released in a liquid medium, as determined by the Tukey test for α < 0.05. Among the three strains, S3 exhibited the greatest resistance to storage in the coated composites prepared at pH 5.5. In both the blended and core-shell composites at pH 3.8 and 5.5, the release times were consistently 48 h. The delayed release of viable cells from these composites may be attributed to the low viability of strains located in the outer capsule layers. The propagation capacity of the S3 strain released from the coated composites could be due to the enhanced protection offered by the chitosan layer, which reduced the porosity of the alginate capsules by approximately 50%, as shown in Figure 6. The *Streptomyces* sp. strain released from the various encapsulations showed no signs of viability, even after 48 h of shaking. Notably, during the biomass release process, both blended and alginate capsules collapsed within the first 24 h of shaking, indicating low mechanical resistance. The viable mycelium released from the different composites was subsequently analyzed for its ability to produce indole acetic acid and solubilize phosphates, two key functions crucial for their application in the agricultural sector.

### 3.8. FTIR Spectra

**Figure 9:**
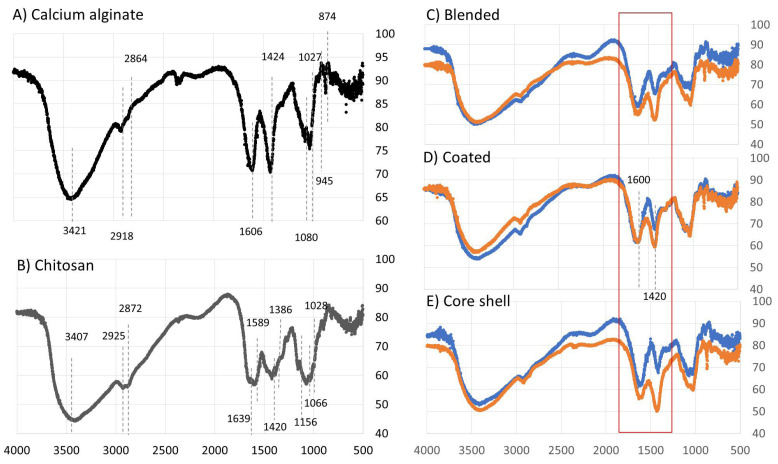
FTIR spectra of calcium alginate (**A**) and chitosan (**B**) capsules and their composites: blended (**C**), coated (**D**), and core shell (**E**), produced when the chitosan was solubilized at a pH of 3.8 (blue line) and 5.5 (orange line).

Figure 9 presents the FTIR spectra of alginate and chitosan gels, as well as those of the alginate–chitosan composites prepared with solubilized chitosan at pH 3.8 and 5.5. The obtained spectra are consistent with those previously reported in the literature [47,48,49,50]. In the FTIR spectrum of calcium alginate, the signals within the range of 3000–3600 cm^−1^ correspond to the stretching vibrations of O–H bonds, while the signals in the range of 2923–2857 cm^−1^ are attributed to the symmetric and asymmetric stretching of C-H bonds. These latter bands are characteristic of polysaccharides and are thus also present in the spectra of both chitosan and the composites. The signals at 1606 cm^−1^ and 1429 cm^−1^ are assigned to the asymmetric and symmetric stretching vibrations of the carboxylate group (COO^−^), respectively. The bands at 1080 cm^−1^ and 945 cm^−1^ are associated with the stretching vibration of the C–O bond in the pyranosyl ring and the C–O stretching in the deformation of the C–C–H and C–O–H bonds.

In the FTIR spectrum of chitosan, the band around 3417 cm^−1^ corresponds to the stretching vibrations of N–H and O–H bonds. The bands at 1639 cm^−1^ and 1386 cm^−1^ represent the C=O stretching signal of amide I and the C–N stretching signal of amide III, respectively. The band at 1589 cm^−1^ is attributed to the bending of the N–H bond in amine II. Signals at 1420 cm^−1^ and 1385 cm^−1^ correspond to the bending of CH_2_ and the symmetric deformations of CH_3_, respectively. The absorption band at 1156 cm^−1^ is attributed to the asymmetric stretching of the C–O–C bridge, while the bands at 1066 and 1028 cm^−1^ correspond to the stretching of the C–O bond.

Compared with the FTIR spectra of calcium alginate and chitosan, some differences were observed in the spectra of the composites, particularly in the characteristic signals of the biopolymers that compose them. Notably, the signal displacement from 1639 cm^−1^ to 1617 cm^−1^, attributed to the stretching of amide I, stands out. Other changes include the reduction in the intensity of signals at 1386, 1320, and 1153 cm^−1^, associated with the symmetric deformations of CH_3_, the C–N stretching of amide III, and the asymmetric stretching of the C–O–C bridge, respectively. According to the literature, the decrease in or disappearance of the signals may result from the protonation of functional groups. At a pH of 3.8, the signal at 1618 cm^−1^ exhibits a higher intensity compared with the signal at 1420 cm^−1^. This relationship in peak intensity is reversed at pH 5.5. According to Singha et al. [13], the increase in the signal at 1420 cm^−1^ indicates a stronger interaction between the NH_3_^+^ and COO^−^ groups, which consequently leads to a decrease in the signal at 1618 cm^−1^, corresponding to the COO^−^ group. This variation in the intensity of these two signals in the FTIR spectrum of the composites suggests that the interaction between chitosan and alginate is more favorable at pH 5.5. This observation agrees with the porosity values presented in Figure 5, where the fresh composites with the lowest porosity are those prepared with chitosan solubilized at pH 5.5.

## 4. Discussion

Increasing biomass production is a key challenge for the commercialization of biofungicides. In the case of actinomycetes, optimal propagation requires specific culture media and carefully controlled environmental conditions. In this study, the carbon, nitrogen, and mineral sources in the M2 medium increased the production of *Streptomyces* sp. by approximately 50% (5.5 g L^−1^) compared with the results reported by Mancera et al. [30], who used a medium based on yeast extract, glucose, and mineral salts. For the actinomycetes S3 and S6, biomass production was 50% higher (8.01 and 8.5 ± 0.5 g L^−1^, respectively) compared with that of *Streptomyces* sp. High yields in the production of actinomycetes using the M2 culture medium could be of commercial interest, particularly since commercial products based on *Streptomyces* spores, such as Actinovate^®^ AG, contain the microbial component at a concentration of 0.0371% *w*/*w* (1 × 10^7^ CFU g^−1^). In the field, this product is applied at a concentration of 0.5 kg ha^−1^ on leaves and 1 kg ha^−1^ on soil.

In liquid culture, actinomycetes produce micellar particles organized into long structures known as filaments. Under conditions of turbulent agitation, strains with a micellar morphology can be damaged, leading to a loss of viability [51]. In this study, the loss of viability of immobilized mycelium in fresh capsules could be attributed to factors such as the immobilization method, the pH used for chitosan solubilization, and the specific characteristics of each actinomycete. Immobilization by extrusion involves dripping the biomass suspended in a biopolymer solution into a gelling solution. When alginate is used as the biopolymer, the drop volume is determined by the size of the pellet formed by the actinomycetes in their micellar form; large pellets can clog the extruder. Forcing these pellets through the extruder can damage the biological material. It is also important to note that chitosan in solution exhibits pseudoplastic behavior [52], so droplet production was only achieved by using a larger-diameter needle and simultaneously increasing the dropping pressure [53].

The total loss of viability of the strains immobilized in chitosan alone was attributed to the increased shear force and the pH of the solutions used, both for chitosan solubilization (pH 3.8) and gelation (pH 14, using a KOH solution) (results not included in this paper) [42]. Although microbial activity has been reported in chitosan, this property depends on factors such as the origin of the chitosan, its concentration, and its degree of acetylation, among other factors [54,55]. In this study, the viability percentages, ranging from 50% to 88% for the strains immobilized in the composites, rule out any significant microbial activity of the chitosan. Additionally, the characteristics of the strains determine their resistance to the different treatments applied in this study, such as the immobilization and dehydration processes [56].

Regarding the resistance of microbial strains to stress factors, Bhowmick et al. [8] observed that *Streptomyces* strains can accumulate intracellular compounds, such as sugars and polyols, that function as osmolytes to help maintain cellular integrity during dehydration. Additional defense mechanisms identified in strains of the same genus include water transport systems that actively regulate cellular water balance, the production of heat shock proteins, and the modification of gene expression. Among the three strains selected for the present study, the ones native to agricultural soil (S3 and S6) demonstrated the highest resistance to the dehydration process, even under air flows of 10 L min^−1^, which was considered the most aggressive condition for the three dehydrated strains. Their greater resistance to dehydration may be attributed to the semi-arid properties of the soil from which they were isolated.

Encapsulation in polymeric materials is a proposed strategy to protect microorganisms from stressful conditions that can lead to their loss of viability [8]. In the case of alginate–chitosan composites, the protective effect has been attributed to the low-porosity reticular structure generated by COO^−^ … NH_3_^+^ bonds during the integration of chitosan into the alginate matrix. As reported in the literature, the structure of this reticular network is determined by various factors, such as the molecular weight of the biopolymers, the degree of chitosan deacetylation, and the pH selected for biopolymer solubilization [13,41,57]. In the present work, with the biopolymers used, increasing the pH from 3.8 to 5.5 resulted in a decrease in the porosity of the fresh capsules for both the coated and core-shell composites. The greater viability of the strains encapsulated in these composites suggests that the lower porosity of the composite may have played an important protective role during the dehydration process.

Regarding the dehydration of biopolymers, Conzatti et al. [58] reported that the dehydration method significantly influences the porosity of the dehydrated biopolymer. In the present study, where dehydration was carried out using an airflow method, the porosity of the encapsulates increased by 20% to 40% in the composites prepared with chitosan at pH 5.5. For composites prepared with chitosan at pH 3.8, significant changes in porosity (up to 20%) were only observed in the blended composites. Although the porosity of the coated composites at pH 5.5 increased by up to 40%, the microbial cells released from these composites exhibited the highest viability percentages.

The analysis of the viability and propagation capacity of the encapsulated strains, dehydrated and stored for 6 months at 6 °C, showed a higher propagation capacity in the native strains released from the coated composites of pH 5.5. In the coated composites of pH 3.8, the propagation capacity of these strains was mainly observed in capsules dehydrated at an airflow of 4 L min^−1^. These findings are consistent with those reported by Albadran et al. [59], who reported that low dehydration rates can help maintain the viability of strains for longer periods.

It is important to mention the crumbling of the alginate capsules and blended composites during the release of the microbial cells in saline water conducted under shaking conditions at 150 rpm. Kulig et al. [60], who studied the mechanical strength and elasticity of gels prepared with different proportions of alginate/chitosan, reported higher mechanical strength in gels with an alginate/chitosan ratio greater than 0.7. Consistent with these findings, our study showed that the coated composites with an alginate/chitosan ratio of 0.8 retained their round structure during the process of releasing microbial strains at 150 rpm, while the blended composites with an alginate/chitosan ratio of 0.52 collapsed, as evidenced by the increased turbidity of the water used to collect the microbial cells.

## 5. Conclusions

Vegetative cells of actinomycetes native to agricultural soils were successfully immobilized with minimal viability loss in alginate–chitosan composites, categorized as blended, coated, and core-shell. Among these, the coated composite offered the highest level of protection, with microbial cells retaining 89 ± 5% viability under an airflow of 4 L min^−1^. This enhanced protective effect is attributed to the low porosity (~50%) of the fresh polymeric matrices during the dehydration process. The sustained viability and propagation capacity of the vegetative cells released from the dehydrated capsules could be explained by the nutrient availability, facilitated by the increased porosity of the polymeric capsules post-dehydration.

The alginate–chitosan polymeric complex has demonstrated effectiveness in encapsulating organic materials with diverse characteristics. In this study, the immobilization of vegetative actinomycete cells in coated alginate–chitosan composites, followed by dehydration with airflow at environmental temperature, resulted in polymeric matrices with properties useful for preserving the biological material and ensuring its effective application in the sustainable production of crops.

## Figures and Tables

**Table 1:** Experimental procedure to produce the calcium alginate–chitosan composites.

Composite	Step 1	Step 2
Blended	A 100 mL solution of sodium alginate (1.5% *w*/*v*) with suspended biomass was dripped into 400 mL of a solution ^1^ containing chitosan (0.8% *w*/*v*, pH 3.8) and CaCl_2_ (0.2 M) using a peristaltic pump and hypodermic needles (31 G × 8 mm and 20 G × 9.5 mm). The mixture was allowed to gel at 150 rpm for 60 min.	
Coated	A 100 mL solution of sodium alginate (1.5% *w*/*v*) with suspended biomass was dripped into 400 mL of 0.1 M CaCl_2_ solution using a peristaltic pump and hypodermic needles (31 G × 8 mm and 20 G × 9.5 mm). The mixture was allowed to gel at 150 rpm for 60 min.	Calcium alginate capsules with immobilized biomass were immersed for 180 min at 150 rpm in 400 mL of a 0.8% (*w*/*v*) chitosan solution. This solution was prepared using distilled water acidified with 0.1% (*w*/*v*) acetic acid to a pH of 3.8.
Core-Shell	A 100 mL solution of sodium alginate (1.5% *w*/*v*) with suspended biomass was dripped into 400 mL of 0.03 M CaCl_2_ solution using a peristaltic pump and hypodermic needles (31 G × 8 mm and 20 G × 9.5 mm). The mixture was allowed to gel at 150 rpm for 60 min.	Calcium alginate capsules with immobilized biomass were immersed for 120 min at 150 rpm in 400 mL of a solution ^1^ containing chitosan (0.8% *w*/*v*, pH 3.8) and CaCl_2_ (0.2 M)
Control	See Section 2.3	

^1^ Chitosan was initially solubilized in distilled water acidified with 0.1% (*w*/*v*) acetic acid to achieve a pH of 3.8. Calcium chloride was then added to the chitosan solution to reach a final concentration of 0.2 M. The solution was sterilized at 121 °C and 9.8 kPa for 15 min.

**Table 2:** Mean diameter and sphericity factor of capsules containing immobilized biomass prepared with sodium alginate, chitosan, and their composites (chitosan solubilized at pH = 3.8 and 5.5 in composites).

Polymer Matrix	Mean Diameter (mm)			Sphericity Factor		
pH	3.8	5.5	6.5	3.8	5.5	6.5
Calcium alginate			2.4 ± 0.0 ^b^			0.059 ± 0.044 ^ab^
Chitosan	4 ± 0.7 ^d^			0.045 ± 0.029 ^a^		
Composites						
Blended	2.6 ± 0.1 ^c^	2.5 ± 0.2 ^c^		0.080 ± 0.062 ^ab^	0.073 ± 0.042 ^ab^	
Coated	2.1 ± 0.1 ^a^	2.3 ± 0.1 ^a^		0.112 ± 0.040 ^b^	0.077 ± 0.056 ^b^	
Core-shell	2.7 ± 0.1 ^c^	2.5 ± 0.1 ^c^		0.067 ± 0.036 ^ab^	0.080 ± 0.048 ^ab^	

Two-way MANOVA. Capsule diameters with the same letter (a–d) do not significantly differ (Tukey’s test for an α = 0.05; Shapiro–Wilk normality test).

**Table 3:** Percentage (±SD) (%*w*/*w*) of chitosan incorporated into alginate when solubilized in pH 3.8 and 5.5 solvents, and the corresponding alginate/chitosan ratio (*w*/*w*).

Composite Type	Chitosan in Capsules (%) (%*w*/*w*)		Alginate/Chitosan Ratio (*w*/*w*)	
	pH = 3.8	pH = 5.5	pH = 3.8	pH = 5.5
Blended	90.4 ± 3.3 ^a^	71.7 ± 3.2 ^a^	0.52	0.65
Coated	64.4 ± 1.5 ^b^	59 ± 3 ^b^	0.73	0.8
Core-shell	74 ± 2 ^b^	61.6 ± 2.5 ^b^	0.64	0.76

One way ANOVA (*n* = 12). The normality requirement was met with the Shapiro–Wilk test. Tukey’s test for α < 0.05: (F(pH) = F= 30.827, df = 1, *p* = 0.001, χ^2^ = 0.837; F(composite) = F= 27.516, df = 1, *p* = 0.001, χ^2^ = 0.902). Markers labeled with the same letter do not significantly differ.

**Table 4:** Biomass production * (g g^−1^ dehydrated capsules) by actinomycete strains released from calcium alginate capsules, and alginate–chitosan composites at 24 and 48 h. Bold values indicate viable biomass released at 24 h.

pH	Strain	Calcium Alginate			Blend			Coated			Core-Shell		
	Airflow (L min^−1^) →	fresh	4	10	fresh	4	10	fresh	4	10	fresh	4	10
3.8	*Streptomyces*												
	S3				11.9 ± 0.5	0.5 ± 0.1			10.3 ± 0.6	9.3 ± 0.4		12 ± 0.7	13.8 ± 1.8
	S6								5.4 ± 0.2			5.6 ± 0.1	
5.5	*Streptomyces*												
	S3				8.5 ± 0.4	**8.9 ± 0.6**	12.9 ± 0.6	**9.4 ± 0.5**	**9.6 ± 0.1**	**8.1 ± 0.1**	9.1 ± 0.5	**8.6 ± 0.1**	8.9 ± 0.1
	S6							**9.1 ± 0.5**	5.9 ± 0.5	0.3 ± 0.1			
6.5	*Streptomyces*												
	S3	8.9 ± 0.7	9 ± 0.1	16.5 ± 1.1									
	S6	**10.9 ± 0.6**	8.5 ± 0.4	14.1 ± 0.4									

* ANOVA. The propagation of strains released with shaking in liquid medium showed significant differences at α < 0.05 (Tukey test) concerning strain type (F = 2316.2419; df = 1; *p* < 0.0001; χ^2^ = 0.982), pH (F = 432.785; df = 1; *p* < 0.0001; χ^2^ = 0.912), composite type (F = 90.918; df = 2; *p* < 0.0001; χ^2^ = 0.812), and airflow (F = 18.45; df = 2; *p* < 0.0001; χ^2^ = 0.468).

## Data Availability

The original contributions presented in the study are included in the article/Supplementary Materials, further inquiries can be directed to the corresponding author/s.

## Supplementary Materials

The following supporting information can be downloaded at: https://www.mdpi.com/article/10.3390/polym16192691/s1, Figure S1: Colonial morphology of actinomycetes S3 and S6, observed under a microscope (10X) (left side of the figure) and in a Petri dishes (Czapek agar) (right side of the figure).
