# Supplementary material for: Influence of Chitosan on the Viability of Encapsulated and Dehydrated Formulations of Vegetative Cells of Actinomycetes
Source: Polymers (Basel). 2024 Sep 24;16(19):2691. doi: 10.3390/polym16192691 (PMC11478721; doi:10.3390/polym16192691)
Supplement: Supplementary file 1 [file polymers-16-02691-s001.zip › polymers-3165575-supplementary.pdf]

Supplementary figures

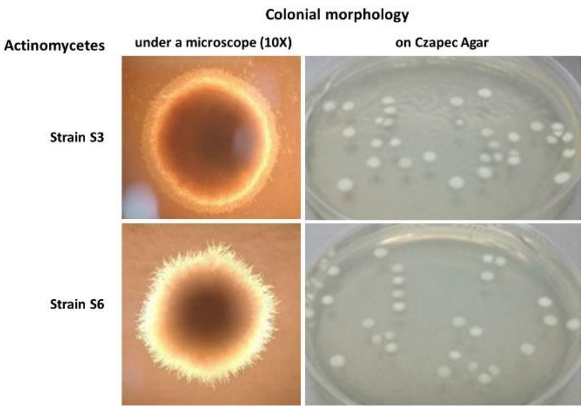

Figure S1. Colonial morphology of actinomycetes S3 and S6, observed under a microscope (10X) (left side of the figure) and in a Petri dishes (Czapeck agar) (right side of the figure).
